# Supplementary material for: Identification of photoperiod-induced specific miRNAs in the adrenal glands of Sunite sheep (Ovis aries)
Source: Front Vet Sci. 2022 Jul 22;9:888207. doi: 10.3389/fvets.2022.888207 (PMC9354845; doi:10.3389/fvets.2022.888207)
Supplement: Supplementary file 1 [file Data_Sheet_1.ZIP › Supplementary file/miRDeep2 file.zip!/miRDeep2 file/Know_Structure/SP42A1/oar-mir-152.pdf]

miRBase precursor : oar-mir-152
Total read count : 15600
oar-miR-152 read count : 15600
remaining reads : 0

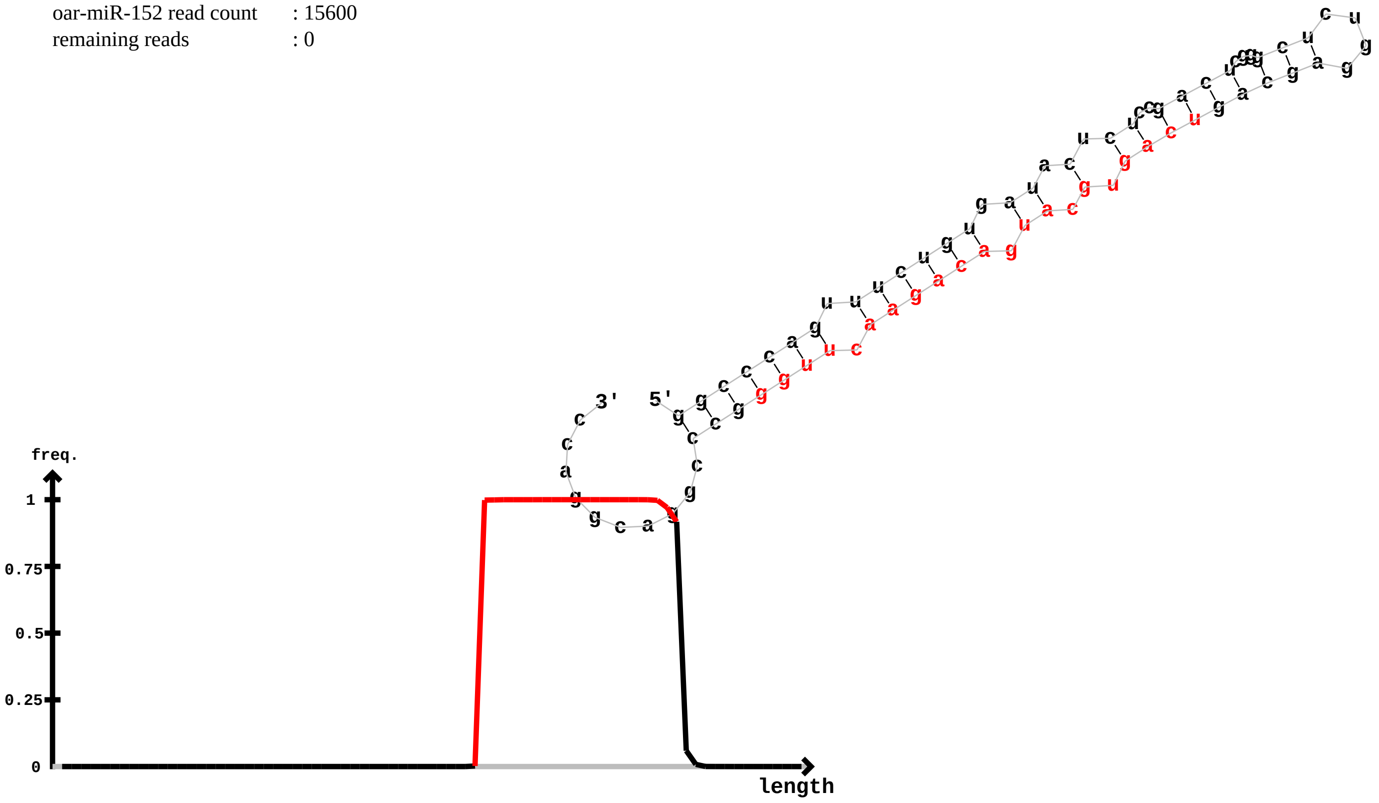

oar-miR-152

```
5'- ggcccaguuucugugauacucuccgacucgggcucuggagcagucagugcaugacagaacuugggcccggacggacc   -3'    exp
    (((((((.((((((.((.(.((..((((...(((....))))))))).).)).)))))).))))))).........    reads      mm         sample
    ..........................................gucagugcaugacagaacuu...............    2          0          seq
    ..........................................gucagugcaugacagaacuug..............    5          0          seq
    ..........................................gucagugcaugacagaacuugg.............    23         0          seq
    ..........................................gucagugcaugacagaacuuggg............    2          0          seq
    ...........................................ucagugcauCacagaac.................    1          1          seq
    ...........................................ucagugcaugacagaac.................    2          0          seq
    ...........................................ucagugcauCacagaacu................    5          1          seq
    ...........................................ucagugcaugacagaacu................    30         0          seq
    ...........................................ucagugcaugacUgaacu................    1          1          seq
    ...........................................ucagugcaugacagaacuu...............    406        0          seq
    ...........................................ucagugcauCacagaacuu...............    12         1          seq
    ...........................................ucagugcaugacagaacuug..............    822        0          seq
    ...........................................ucagugAaugacagaacuug..............    4          1          seq
    ...........................................ucagugcaugacagaacuugg.............    13340      0          seq
    ...........................................ucagugcaugacagaacuuggg............    797        0          seq
    ...........................................ucagugcaugacagaacuugggc...........    121        0          seq
    ...........................................ucagugcaugacagaacuugggcc..........    4          0          seq
    ............................................cagugcaugacagaacuugg.............    9          0          seq
    .............................................agugcaugacagaacuug..............    1          0          seq
    .............................................agugcaugacagaacuugg.............    7          0          seq
    .............................................agugcaugacagaacuugA.............    4          1          seq
    ...............................................ugcaugacagaacuugA.............    1          1          seq
    .................................................caugacagaacuugggcccg........    1          0          seq
```
